# Is the superbug fungus really so scary? A systematic review and meta-analysis of global epidemiology and mortality of *Candida auris*

**DOI:** 10.1186/s12879-020-05543-0

**Published:** 2020-11-11

**Authors:** Jingjing Chen, Sufei Tian, Xiaoxu Han, Yunzhuo Chu, Qihui Wang, Baosen Zhou, Hong Shang

**Affiliations:** 1 Department of Laboratory Medicine, the First Affiliated Hospital of China Medical University, No. 155, Nanjing North Street, Heping District, Shenyang, 110001 Liaoning China; 2 National Clinical Research Center for Laboratory Medicine, the First Affiliated Hospital of China Medical University, Shenyang, 110001 China; 3 Collaborative Innovation Center for Diagnosis and Treatment of Infectious Diseases, 79 Qingchun Street, Hangzhou, 310003 China; 4 Department of Clinical Epidemiology and Center of Evidence-Based Medicine, the First Hospital of China Medical University, Shenyang, 110001 China

**Keywords:** *Candida auris*, Case count, Drug resistance, Mortality, Bloodstream infection, Clade

## Abstract

**Background:**

*Candida auris* is a new pathogen called “superbug fungus” which caused panic worldwide. There are no large-scale epidemiology studies by now, therefore a systematic review and meta-analysis was undertaken to determine the epidemic situation, drug resistance patterns and mortality of *C. auris*.

**Methods:**

We systematically searched studies on the clinical report of *Candida auris* in Pubmed, Embase and Cochrane databases until October 6, 2019. A standardized form was used for data collection, and then statics was performed with STATA11.0.

**Results:**

It showed that more than 4733 cases of *C. auris* were reported in over 33 countries, with more cases in South Africa, United States of America, India, Spain, United Kingdom, South Korea, Colombia and Pakistan. *C. auirs* exhibited a decrease in case count after 2016. Clade I and III were the most prevalent clades with more cases reported and wider geographical distribution. Blood stream infection was observed in 32% of the cases, which varied depending on the clades. Resistance to fluconazole, amphotericin B, caspofungin, micafungin and anidulafungin in *C. auris* were 91, 12, 12.1, 0.8 and 1.1%. The overall mortality of *C. auris* infection was 39%. Furthermore, subgroup analyses showed that mortality was higher in bloodstream infections (45%), and lower in Europe (20%).

**Conclusions:**

Over 4000 cases of *C. auris* were reported in at least 33 countries, which showed high resistance to fluconazole, moderate resistance to amphotericin B and caspofungin, high sensitivity to micafungin and anidulafungin. The crude mortality for BSI of *C. auris* was 45% which was similar to some drug-resistant bacteria previously reported. In conclusion, *C. auris* displayed similar characteristics to some drug resistance organisms. This study depicts several issues of *C. auris* that are most concerned, and is of great significance for the clinical management.

**Supplementary Information:**

The online version contains supplementary material available at 10.1186/s12879-020-05543-0.

## Background

*Candida auris* is a recently emerging nosocomial pathogen which was initially described in Japan in 2009 and then reported in over 30 countries worldwide afterwards [1, 2]. *C. auris* is usually resistant to several drugs, such as fluconazole, voriconazole, amphotericin B. However, resistance rate varies between studies. According to the genome sequences, *C. auris* isolates were divided into four clades that were separated by tens of thousands of SNPs: Clade I (South Asian), Clade II (East Asian), Clade III (South African), Clade IV (South American) [3]. Besides, a potential Clade V was found in Iran recently [4].

*C. auris* can infect or colonize in humans, especially the low-immunity patients in the intensive care unit. Infection and colonization of *C. auris* are associated with varied treatment strategies and clinical outcomes, so they should be differentiated. Blood stream infections (BSI) are the most common infections with serious outcomes. Overall mortality of *C. auris* and that for patients with BSI may be as high as 59 and 68% respectively [3]. Nevertheless, other studies reported different data.

Due to its transmissibility, multidrug resistance and severe outcomes, *C. auris* is called “superbug fungus”. Due to the low incidence of *C. auris*, no large-scale epidemiology studies were reported by now. Therefore, a comprehensive study was needed to summarize the global epidemiology of *C. auris*. In this present study, we performed a systematic review and meta-analysis to estimate the case count, drug resistance and mortality of *C. auris*. Moreover, factors that may affect the mortality such as BSI, clade and drug resistant patterns of *C. auris* were also analyzed.

## Methods

### Search strategies and study selection

This systematic review and meta-analysis was carried out according to Preferred Reporting Items for Systematic Reviews and Meta-Analyses (PRISMA) guidelines. We systematically searched Pubmed, Embase and Cochrane databases from inception until October 6, 2019 with the only keyword “*Candida auris*”. Additional studies were obtained by screening the references of eligible studies. Besides, we also searched the websites of Centers for Disease Control and Prevention (CDC), European Centers for Disease Control and Prevention (ECDC) and Public Health England (PHE). Three objectives of this study were case count, drug resistance and mortality of *C. auris*. Since CDC has established breakpoints for fluconazole, amphotericin B, caspofungin, micafungin and anidulafungin in *C. auris* (fluconazole ≥32, amphotericin B ≥ 2, anidulafungin ≥4, caspofungin ≥2 and micafungin ≥4 deemed to be drug-resistant), only these drugs were analyzed in this present study.

### Inclusion and exclusion criteria

All study formats met the following criteria were included in the meta-analysis: 1) Studies that reported the information of case count, drug resistance and mortality of *C. auris*, with no limit regarding the diagnostic test used for detecting *C. auris*. 2) Studies that provided the case count of patients with *C. auris*, number of resistant isolates/total number of *C. auris* isolates, number of deaths/total number of cases; 3) Studies with sample size larger than 5 for meta-analysis of drug resistance and mortality. While studies met the following criteria were excluded from the analysis: 1) Duplicate studies contained the same patients; 2) For meta-analysis of drug resistance of *C. auris*, studies of which the drug resistance data can’t be reinterpreted according to the CDC breakpoints.

### Data extraction

Title and abstract review of all searched articles was completed by two of the authors (Jingjing Chen and Sufei Tian) to identify relevant studies on the clinical report of *C. auris*. Then full texts of relevant articles were independently reviewed by two of the authors (Xiaoxu Han and Sufei Tian) to determine eligible studies by research objectives. Data in the articles were collected with a standardized form by two of the authors (Jingjing Chen and Xiaoxu Han) independently. Disagreements were discussed by three authors to reach consensus. The following information was extracted: first author’s name, publication year, country, research time, study design, clade, case count, sample type, mortality, drug resistance patterns, methods of drug resistance methods. Drug resistant data were reinterpreted according to the CDC breakpoints.

Quality of the studies included for mortality and drug resistance analysis were assessed by the Agency for Healthcare Research and Quality (AHRQ) checklist (https://www.ncbi.nlm.nih.gov/books/NBK35156/). This 11-item checklist assesses studies in terms of the source of information, inclusion and exclusion criteria, selection of participants, researcher bias, quality assurance, possible confounding variables, handling of missing data, participant response rates, and completeness of data collection. An item would be scored “1” for “YES” and scored “0” for “NO” or “UNCLEAR”. Article quality was classified as follows: low quality = 0–3; moderate quality = 4–7; high quality = 8–11.

### Statistical analysis

The pooled estimate and corresponding 95% confidence interval (CI) were calculated with STATA11.0 software. Statistical heterogeneity was evaluated with Q statistic (*p* < 0.10 indicating statistically significance) and quantified using the I^2^ index. Due to the heterogeneity among studies, all pooled estimates were performed with random-effects model. Furthermore, we did subgroup analyses for mortality stratified by continents, publication year / research year, clade of *C. auris*, sample type (BSI and non-BSI) and drug resistance rate (higher than overall estimate and lower than overall estimate). Moreover, meta-regression was performed to assess risk factors associated with mortality, with variables such as bloodstream infection, clade, fluconazole resistance, amphotericin B resistance, continent, and publication year included into the analysis. Sensitivity analysis was also performed by omission of studies. Begg’s and Egger’s tests were used to assess publication bias, with *p* < 0.05 deemed as statistically significant.

## Results

### Search and identification of eligible studies

As shown in Figure S1, a total of 577 citations were obtained according to the designed search strategy as described in methods. Among them, 97 eligible articles on the clinical report of *C. auris* were selected for further evaluation and 67 studies were included in the meta-analysis. Finally, 57, 21 and 19 studies were enrolled in the analysis for case count, drug resistance and mortality of *C. auris* respectively [1, 4–67].

The publication year of eligible studies ranged from 2009 to 2019. Most studies were observational studies except for two studies which were case-control studies [14, 26]. Detailed characteristics of the eligible articles were summarized in Table S1. The mean quality score of the studies included in the meta-analysis for mortality and drug resistance patterns was 6.2 (range: 4–9), with only one high quality study (Table S2). The main problems of the included articles were lack of information on quality assurance, possible confounding variables, handling of missing data, and completeness of data collection.

### Case count and clade of *C. auris*

A total of 4733 cases of *C. auris* were reported in 33 countries (aligning in descending order: South Africa, United States of America, India, Spain, United Kingdom, South Korea, Colombia, Pakistan, Kenya, Kuwait, China, Russia, Venezuela, Japan, Panama, Israel, Oman, Germany, Brazil, Saudi Arabia, Singapore, France, Australia, Malaysia, Netherlands, Belgium, Norway, Switzerland, United Arab Emirates, Canada, Iran, Greece and Italy) from six continents. The earliest report was in 2009 in Japan, and the earliest isolate of *C. auris* traced back to 1996 in South Korea as showed by several screening experiments [16, 68]. Moreover, an epidemic curve which depicted the case count of *C. auris* by detection year was drawn with studies that contained the detailed information. Notably, this was based on publication data rather than surveillance data. It showed that most cases were detected between 2013 and 2019, peaking in 2016 and decreasing thereafter.

**Fig. 1:**
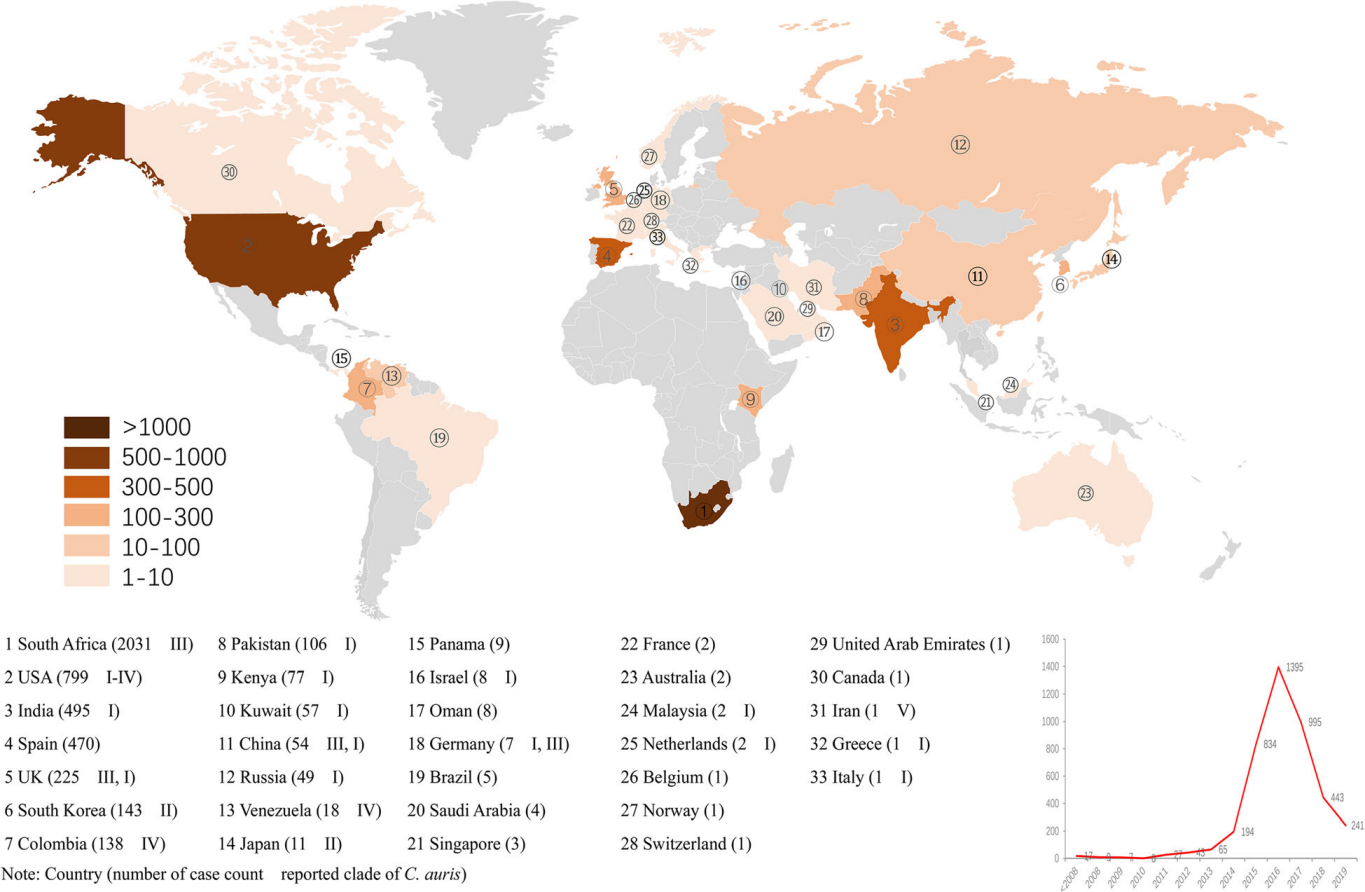
Global reported cases of *C. auris* by country (adapted from Robinson projection map). The reported case count of patients with *C. auris* and clade(s) in different countries were represented in descending order. An epidemic curve showing case count of *C. auris* by year was also portrayed based on publication data

Different clades of *C. auris* were reported to emerge simultaneously from different continents. Four clades of *C. auris* have unique geographical characteristics. Clade I was mainly reported in India, Pakistan, Kuwait, Russia, United States, United Kingdom, Germany, Malaysia, Netherlands, Italy, etc.; And Clade II were mainly in Japan and South Korea. Clade III was mainly found in South Africa, United States, United Kingdom and China, whereas Clade IV mainly distributed in Colombia and Venezuela. Clade I and III were the most prevalent clades which have more reported cases and wider geographical distribution. The case count and clade of *C. auris* stratified by country were shown in Fig. 1.

### Blood stream infection of *C. auris*

**Fig. 2:**
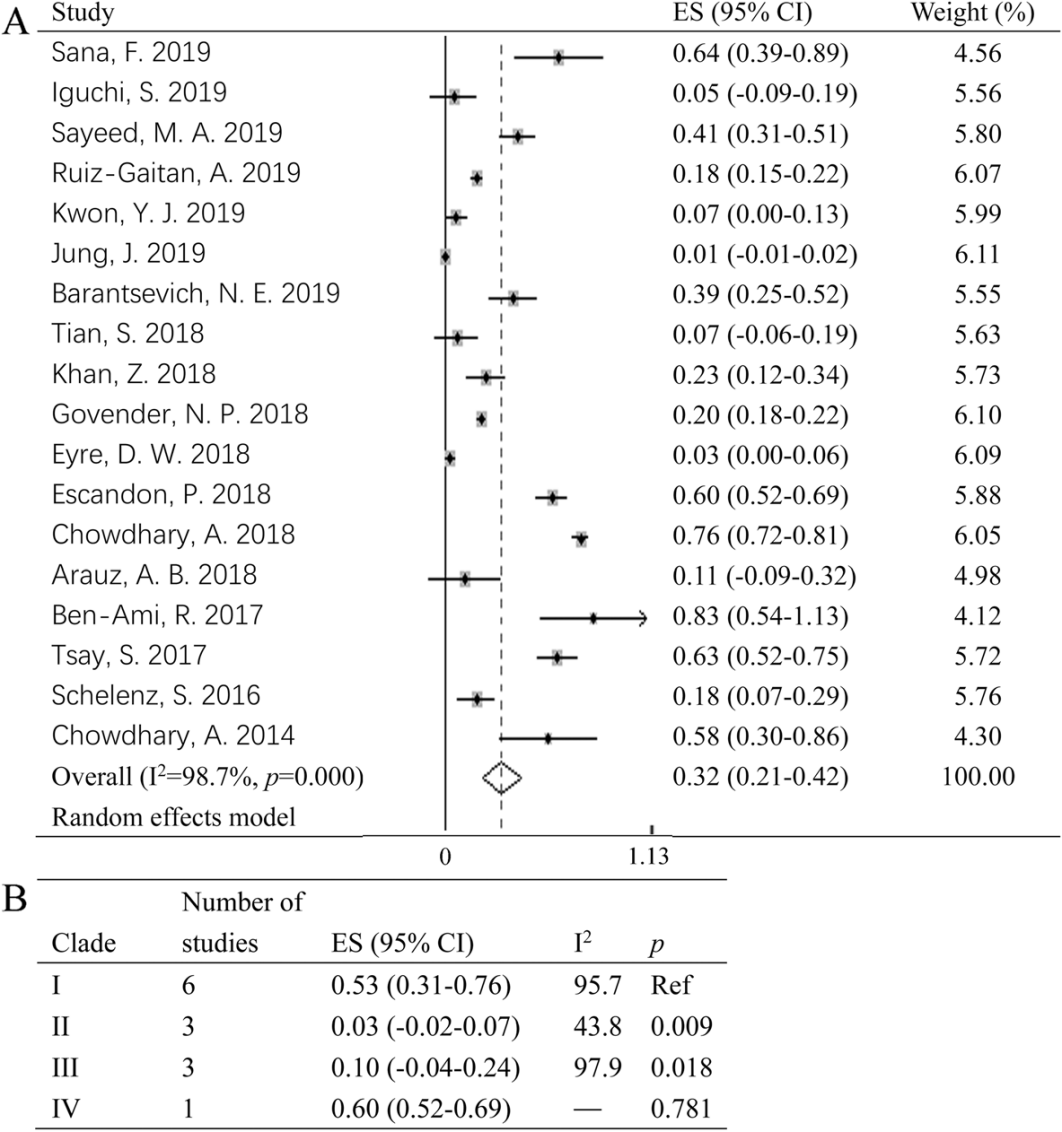
Forest plot on BSI rate of *C. auris* (**a**) and subgroup analysis by clade (**b**). ES: Effect size

Infection and colonization of *C. auris* should be differentiated due to varied clinical significance. However, it is difficult to perform due to unavailable data in the original studies. Therefore, rate of BSI which is the most common and serious infection is analyzed instead. Studies enrolled only the candidaemia patients of *C. auris* were excluded. As shown in Fig. 2a, the frequency of BSI of *C. auris* varied between studies [25, 36, 37], with a pooled rate of blood stream infection of 32% (95% CI: 21–42%). However, heterogeneity (*p* = 0.00, I^2^ = 98.7%) was observed between studies. Subgroup analysis showed that Clade I and Clade IV of *C. auris* has a high percentage of BSI compared to Clade II and Clade III (Fig. 2b). It is worth mentioning that Clade II has a low rate of BSI rate with ear discharge as the main specimen type, which is different from the other clades of *C. auris* [9, 17, 69].

### Drug resistance patterns

**Fig. 3:**
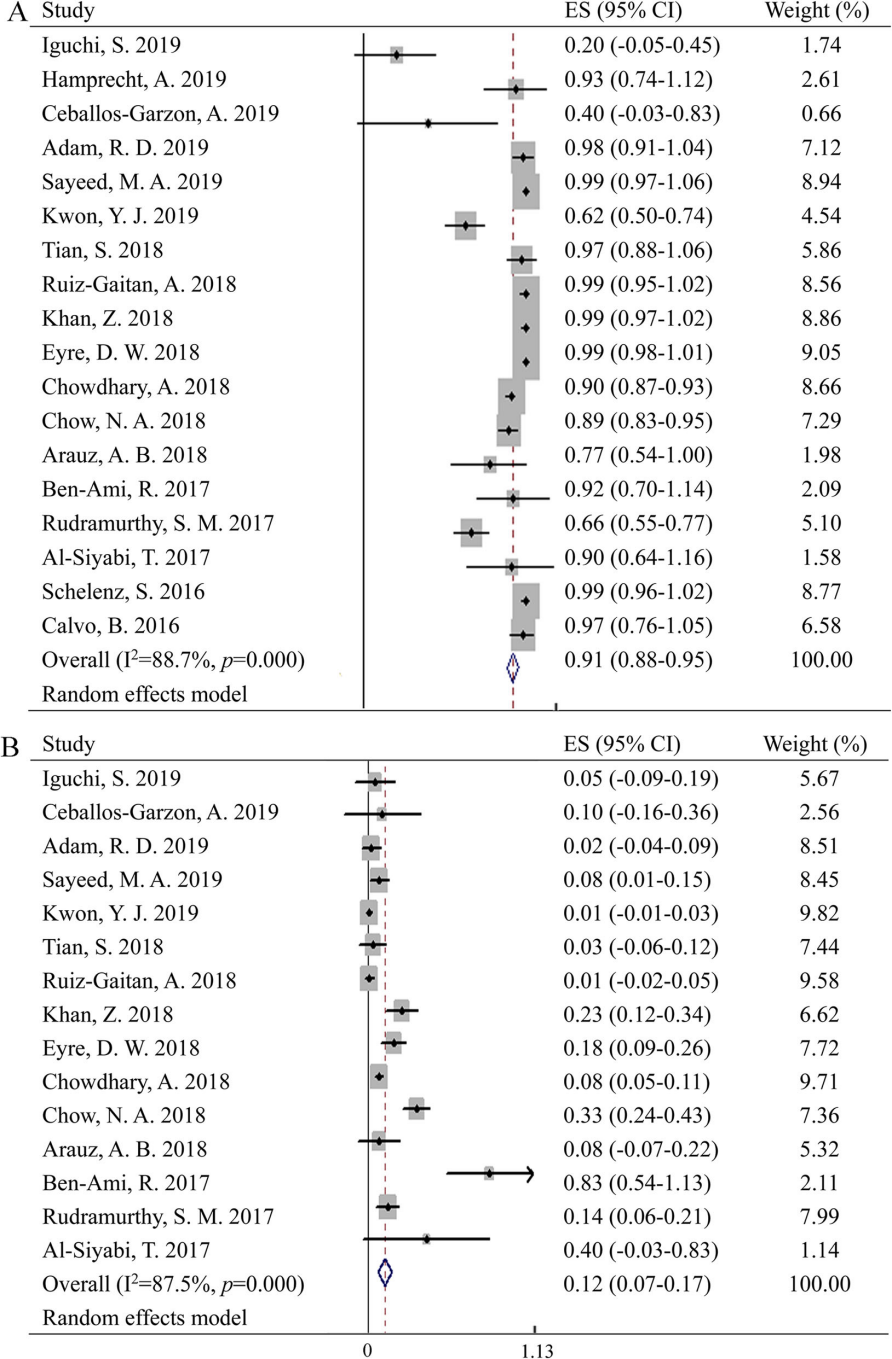
Forest plot on the drug resistance of *C. auris* to fluconazole (**a**) and amphotericin B (**b**)

Meta-analyses of drug resistance were performed with data obtained according to the breakpoints for *C. auris* established by CDC. As shown in Fig. 3, the pooled resistance rate for fluconazole and amphotericin B were 91% (95% CI: 88–95%) and 12% (95% CI: 7–17%) respectively. Yet there was significant heterogeneity between studies. Besides, publication bias was observed for meta-analysis of resistance rate for fluconazole (Figure S2), yet trim and fill method did not get good result.

Meta-analyses for the resistance rate to echinocandins could not be performed as resistance for these drugs are rare in *C. auris*. Descriptive analysis was performed alternatively with frequencies of resistant isolates divided by total isolates. Therefore, resistance rate to caspofungin, micafungin and anidulafungin in *C. auris* were 12.1% (n/*N* = 101/838), 0.8% (n/*N* = 8/927) and 1.1% (n/*N* = 9/840) respectively. However, almost all isolates resistant to caspofungin were from India, with resistance rate of 23.6% (n/*N* = 100/424) for Indian isolates and 0.2% (n/N = 1/414) for non-Indian isolates.

### Mortality of *C. auris*

**Fig. 4:**
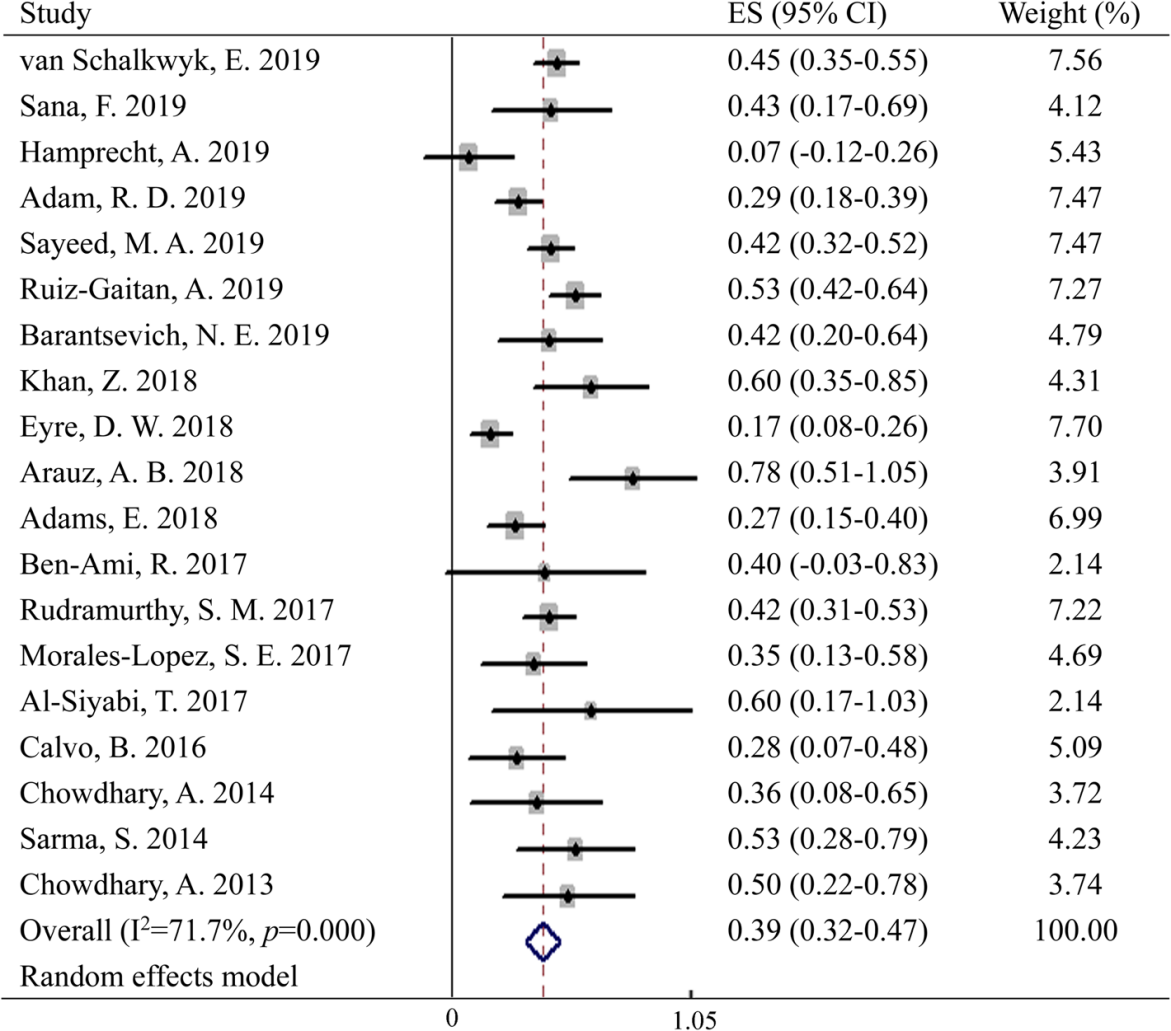
Forest plot on the crude mortality of *C. auris*

The overall crude mortality of *C. auris* ranged from 0 to 78%, with a pooled crude mortality of 39% (95% CI: 32–47%, Fig. 4). While the mortality for BSI of *C. auris* was 45% (95% CI: 39–51%, Figure S3). Negligible publication bias and significant heterogeneity (*p* < 0.05; *I*^2^ = 72%) was observed. Sensitivity analysis indicated that the pooled estimate was quite stable when excluding any of the studies.

Then subgroup analyses were performed to assess factors that may influence the mortality of *C. auris*, such as continent, publication year, clade of *C. auris*, BSI, and resistance to fluconazole, amphotericin B (Table 1). For the subgroup analysis by clade, as most studies contained patients infected with *C. auris* of Clade I, so we stratified the studies as Clade I and non-Clade I, which showed no significant difference. Studies with *C. auris* of Clade II were not included in this analysis as lack of data which may be due to rare death. Notably, mortality of patients with BSI of *C. auris* (45, 95% CI: 39–51%) was higher than that in non-BSI patients (21, 95% CI: 8–33%). Besides, mortality of *C. auris* in Europe (20, 95% CI: 4–37%) was lower than that in Asia (44, 95% CI: 38–51%). However, we did not find associations between mortality and resistance to fluconazole, amphotericin B, clade or publication year.

**Table 1:** Subgroup analyses of pooled mortality

Factors	Group	Number of studies	Pooled crude mortality (95%CI)	I^2^ (%)	*p* value
Bloodstream infection	Yes	15	0.45 (0.39–0.51)	41	**0.002**
	No	5	0.21 (0.08–0.33)	40	Ref
Clade	Clade I	11	0.39 (0.31–0.47)	49	0.343
	Non- Clade I	4	0.31 (0.14–0.48)	83	Ref
FLC resistance	Higher	10	0.29 (0.21–0.38)	60	0.415
	Lower	3	0.49 (0.29–0.70)	70	Ref
AmB resistance	Higher	6	0.29 (0.19–0.40)	45	0.159
	Lower	6	0.43 (0.32–0.53)	62	Ref
Continent	Asia	9	0.44 (0.38–0.51)	0	**0.000**
	America	5	0.43 (0.27–0.59)	78	0.164
	Africa	2	0.37 (0.21–0.53)	81	0.303
	Europe	3	0.20 (0.04–037)	66	Ref
Publication Year	2018–2019	11	0.42 (0.31–0.53)	86	0.769
	2016–2017	5	0.39 (0.30–0.48)	0	0.415
	2013–2015	3	0.47 (0.31–0.63)	0	Ref

*FLC* fluconazole, *AmB* amphotericin B

## Discussion

*C. auris* is a globally spreading yeast with more than 4733 cases reported by now, covering at least 33 countries from six continents. It showed 91% resistance to fluconazole, 12% resistance to amphotericin B, 12% resistance to caspofungin and were highly sensitive to micafungin and anidulafungin. The pooled crude mortality of *C. auris* was 39%, while the mortality of BSI was 45%. Subgroup analyses showed that cases of BSI and from Europe were factors that affected the mortality. This study is helpful for the surveillance and clinical management of *C. auris*.

Although a simple meta-analysis of *C. auris* was performed previously [70], we comprehensively described the epidemic situation and mortality of *C. auris*. Referring to the epidemic situation of *C. auris*, over 4733 cases from 33 countries were reported. However, the actual number of cases was underreported in this study. There may be publication bias and bias based on type of surveillance conducted. First of all, there are countries with *C. auris* cases but not published in literature, such as Thailand, Chile and Bangladesh, Austria and Costa Rica [52, 71]. Secondly, there is bias based on type of surveillance conducted, as screening for *C. auris* may not be adequate in some countries. For instance, although many cases were reported in South Africa and Kenya, the other underdeveloped countries in Africa did not report cases of *C. auris*. Moreover, many patients colonized with *C. auris* which are difficult to identify may be overlooked [9]. This indicates that more intensive surveillance is needed to better understand its epidemic situation. An epidemic curve was drawn using studies with detection time, which showed a peak in 2016 and a fall thereafter. Whether this was a true reduction in case count or a delay in case report needs further follow-up.

As for the clade of *C. auris*, Clade I and Clade III are the geographically prevalent clade, whereas Clade II and Clade VI showed local epidemic. Besides, we found that Clade I and Clade VI of *C. auris* exhibits high BSI rate in comparison with the other clades, which was deemed as severe disease with high mortality. Whether this difference was due to specific genetic features deserves further exploration. Furthermore, as there are genes for mating and meiosis in *C. auris*, sexual recombination can occur with frequent travelling of people with *C. auris*. Consequently, the genome of *C. auris* may become more complicated.

In addition, antifungal resistance patterns were also analyzed. Resistance rate of *C. auris* to fluconazole, amphotericin B, caspofungin, micafungin and anidulafungin were 91, 12, 12.1, 0.8 and 1.1% respectively. It was surprising that Indian isolates showed a resistance rate of 23.6% for caspofungin, which deserves the attention of the clinicians but also needs further validation. Like the other species in the Metschnikowiaceae family (such as *C. haemulonii*), antifungal resistance is common in *C. auris*, limiting the treatment options. Acquired resistance through treatment is another concern which deserves clinicians’ attention and further study [5]. Mutations in ERG11 (Y132F, K143R and F126L) and FKS1 (S639F) play an important role in the drug resistance of fluconazole and echinocandins, which should be detected to guide clinical treatment [72]. Drug resistance to amphotericin B may be inducible and transient, nonetheless the mechanisms are not well understood yet. Moreover, genomic insights and analyses of gene expression showed that genes associated with oligopeptide and ABC transporters, iron transporters, glycophosphatidylinositol-anchored proteins, etcmay be involved in drug resistance of *C. auris* [73, 74].

The pooled crude mortality of *C. auris* infection was 39%, with an overall mortality of BSI of 45%. Previous meta-analysis indicated that the mortality of candidemia in Europe was 38% [75]. Moreover, the mortality of *C. auris* was also compared with other drug-resistant organisms, which spread in similar ways in healthcare centers. A meta-analysis showed that the mortality of patients infected with multidrug-resistant *Pseudomonas aeruginosa* was 44.6% [76]. Besides, the overall mortality of BSIs of vancomycin resistant *Staphylococcus aureus* and carbapenem-resistant *Klebsiella pneumoniae* were 26.8 and 54.3% respectively [77, 78]. This indicates that the mortality of *C. auris* candidemia was a little higher than the other candidemia and similar to that of some drug-resistant bacterial BSIs.

There was heterogeneity between studies, so we investigated factors that may affect the mortality of *C. auris* infection, such as clade, BSI, drug resistance, continent and publication year. Results showed that the mortality of BSI of *C. auris* was higher than that of non-BSI. Besides, the mortality reported in Europe was lower than that in Asia. This indicated that types of infection and continent were factors for significant heterogeneity. In addition, mortality at any time rather than 30-day mortality, clades of *C. auris*, study designs may be the causes of heterogeneity. Reasons explaining for lower mortality in Europe may be as follows: (1) high percentage of non-BSI [10, 19, 26]; (2) better healthcare systems in developed countries with more intensive surveillance and rational treatment.

Although this study was a comprehensive analysis, some limitations should be noted. Firstly, there was an underestimation in case count of *C. auris* due to publication bias and bias based on type of surveillance conducted. What’s more, most studies included were observational studies, crude mortality rather than attributable mortality was analyzed. Furthermore, significant heterogeneity was observed between studies, well-designed case-control studies should be carried out to estimate the resistance patterns and mortality of *C. auris* accurately.

*C. auris* is an emerging pathogen covering over 33 countries, which may have a decrease in case count after 2016. It showed high resistance to fluconazole, moderate resistance to amphotericin B, and high sensitivity to echinocandins. The crude mortality for BSI of *C. auris* was 45% which was similar to some drug-resistant bacteria previously reported. In summary, *C. auris* displayed similar characteristics to some drug resistance organisms. *C. auris* may not be so scary, yet it should not be underestimated, intensive prevention and control should be taken.

## Supplementary Information

**Additional file 1: Table S1** Characteristics of included studies.

**Additional file 2: Table S2** Quality assessment of the studies included in the meta-analysis for mortality and drug resistance patterns.

**Additional file 3: Figure S1** Flowchart showing study search and selection.

**Additional file 4: Figure S2** Funnel plot for BSI rate of *C. auris* (A), drug resistance of *C. auris* to fluconazole (B) and amphotericin B (C), crude mortality (D).

**Additional file 5: Figure S3** Forest plot on the crude mortality for BSI of *C. auris*.

## Data Availability

The datasets used and/or analyzed of the current study are available from the corresponding author on request.

## Abbreviations

BSI: Bloodstream infections
CDC: Centers for Disease Control and Prevention
ECDC: European Centers for Disease Control and Prevention
PHE: Public Health England
AHRQ: Agency for Healthcare Research and Quality
CI: Confidence interval
