# Supplementary material for: Is the superbug fungus really so scary? A systematic review and meta-analysis of global epidemiology and mortality of Candida auris
Source: BMC Infect Dis. 2020 Nov 11;20:827. doi: 10.1186/s12879-020-05543-0 (PMC7656719; doi:10.1186/s12879-020-05543-0)
Supplement: Supplementary file 1 — Additional file 1: Table S1 Characteristics of included studies. [file 12879_2020_5543_MOESM1_ESM.docx]

Table S1 Characteristics of included studies

| ID | Author | Publication Year | Study characteristics | Research time | Country | Analysis for case count | Analysis for drug resistance | Analysis for mortality |
| --- | --- | --- | --- | --- | --- | --- | --- | --- |
| 1 | US CDC | 2019 | Surveillance report | -2019 | USA | Yes |  |  |
| 2 | Bajpai, V. [1] | 2019 | Case serials | 2017-2018 | India | Yes |  |  |
| 3 | Department of Health, HongKong | 2019 | Surveillance report | 2019 | China | Yes |  |  |
| 4 | van Schalkwyk, E. [2] | 2019 | Epidemiologic survey | 2016-2017 | South Africa | Yes |  | Yes |
| 5 | Chibabhai, V. [3] | 2019 | Single-center retrospective study | 2015-2018 | India | Yes |  |  |
| 6 | Sana, F. [4] | 2019 | Outbreak report | 2017-2018 | Pakistan | Yes |  | Yes |
| 7 | Iguchi, S. [5] | 2019 | Review | 1997-2019 | Japan | Yes | Yes |  |
| 8 | Hamprecht, A. [6] | 2019 | Case serials | 2015-2017 | Germany |  | Yes | Yes |
| 9 | Ceballos-Garzon, A. [7] | 2019 | Single-center prospective study | 2013-2015 | Colombia | Yes | Yes |  |
| 10 | Adam, R. D. [8] | 2019 | Single-center retrospective study | 2011-2016 | Kenya | Yes | Yes | Yes |
| 11 | Sayeed, M. A. [9] | 2019 | Single-center retrospective study | 2014–2017 | Pakistan | Yes | Yes | Yes |
| 12 | Ruiz-Gaitan, A. [10] | 2019 | Prospective, case-controlled study | 2016-2018 | Spain | Yes |  | Yes |
| 13 | Park, J. Y. [11] | 2019 | Case serials | 2016-2018 | USA | Yes |  |  |
| 14 | Kwon, Y. J. [12] | 2019 | Multi-center retrospective study | 1996-2018 | Korea | Yes | Yes |  |
| 15 | Jung, J. [13] | 2019 | Retrospective study | 2016-2018 | Korea | Yes |  |  |
| 16 | Escandon, P. [14] | 2019 | Multi-center retrospective study | 2016 | Colombia | Yes |  |  |
| 17 | Barantsevich, N. E. [15] | 2019 | Single-center retrospective study | 2016-2017 | Russia | Yes | Yes | Yes |
| 18 | Tian, S. [16] | 2018 | Single-center retrospective study | 2016-2017 | China | Yes | Yes |  |
| 19 | Ruiz-Gaitan, A. [17] | 2018 | Outbreak research | 2016-2017 | Spain |  | Yes |  |
| 20 | Kohlenberg, A. [18] | 2018 | Outbreak research | 2013-2017 | Spain, UK, Germany, France, Belgium, Norway | Yes |  |  |
| 21 | Khan, Z. [19] | 2018 | Multi-center retrospective study | 2014-2017 | Kuwait |  |  | Yes |
| 22 | Khan, Z. [20] | 2018 | Multi-center retrospective study | 2014 -2017 | Kuwait | Yes | Yes |  |
| 23 | Govender, N. P. [21] | 2018 | Multi-center retrospective study | 2012-2016 | South Africa | Yes |  |  |
| 24 | Eyre, D. W. [22] | 2018 | Case-control study | 2015-2017 | UK |  | Yes | Yes |
| 25 | Escandon, P. [23] | 2018 | Surveillance report | 2015-2017 | Colombia | Yes |  |  |
| 26 | Chowdhary, A. [24] | 2018 | Multi-center retrospective study | 2009-2017 | India | Yes | Yes |  |
| 27 | Chow, N. A. [25] | 2018 | Molecular epidemiology survey | 2013-2017 | USA |  | Yes |  |
| 28 | Arauz, A. B. [26] | 2018 | Case serials | 2016 | Panama | Yes | Yes | Yes |
| 29 | Adams, E. [27] | 2018 | Retrospective study | 2013-2017 | USA |  | Yes | Yes |
| 30 | Ben-Ami, R. [28] | 2017 | Case serials | 2014-2015 | Israel | Yes | Yes | Yes |
| 31 | Rudramurthy, S. M. [29] | 2017 | Multi-center retrospective study | 2011-2012 | India | Yes | Yes | Yes |
| 32 | Morales-Lopez, S. E. [30] | 2017 | Case serials | 2016 | Colombia |  | Yes | Yes |
| 33 | Al-Siyabi, T. [31] | 2017 | Case serials | 2016-2017 | Oman | Yes | Yes | Yes |
| 34 | Schelenz, S. [32] | 2016 | Outbreak research | 2015-2016 | UK |  | Yes |  |
| 35 | Prakash, A. [33] | 2016 | Retrospective study | 2010-2014 | India, South Africa, Brazil, Korea，Japan | Yes |  |  |
| 36 | Calvo, B. [34] | 2016 | Case serials | 2012-2013 | Venezuela | Yes | Yes | Yes |
| 37 | Chowdhary, A. [35] | 2014 | Case serials | 2011-2013 | India |  |  | Yes |
| 38 | Sarma, S. [36] | 2013 | Case serials | 2011 | India | Yes |  | Yes |
| 39 | Chowdhary, A. [37] | 2013 | Case serials | 2009-2011 | India |  |  | Yes |
| 40 | Ding, C. H. [38] | 2019 | Case report |  | Malaysia | Yes |  |  |
| 41 | Crea, F. [39] | 2019 | Case report | 2019 | Italy | Yes |  |  |
| 42 | Vogelzang, E. H. [40] | 2019 | Case report |  | Nethrelands | Yes |  |  |
| 43 | Supreeth, S. [41] | 2019 | Case report |  | Oman | Yes |  |  |
| 44 | Stathi, A. [42] | 2019 | Case report | 2018 | Greece | Yes |  |  |
| 45 | Elsawy, A. [43] | 2019 | Case report |  | Saudi Arabia | Yes |  |  |
| 46 | Tang, H. J. [44] | 2019 | Case report | 2017 | China | Yes |  |  |
| 47 | O'Connor, C. [45] | 2019 | Case report | 2018-2019 | UK | Yes |  |  |
| 48 | Abastabar, M. [46] | 2019 | Case report | 2017 | Iran | Yes |  |  |
| 49 | Heath, C. H. [47] | 2019 | Case report | 2015 | Austrialia | Yes |  |  |
| 50 | Wang, X. [48] | 2018 | Case report | 2018 | China | Yes |  |  |
| 51 | Tan, Y. E. [49] | 2018 | Case report |  | Singapore | Yes |  |  |
| 52 | Riat, A. [50] | 2018 | Case report |  | Switzerland | Yes |  |  |
| 53 | Pekard-Amenitsch, S. [51] | 2018 | Case report |  | Australia | Yes |  |  |
| 54 | Parra-Giraldo, C. M. [52] | 2018 | Case report | 2013-2015 | Colombia | Yes |  |  |
| 55 | Mohd Tap, R. [53] | 2018 | Case report |  | Malaysia | Yes |  |  |
| 56 | Chen, Y. [54] | 2018 | Case report |  | China | Yes |  |  |
| 57 | Belkin, A. [55] | 2018 | Case report | 2017 | Israel | Yes |  |  |
| 58 | Alatoom, A. [56] | 2018 | Case report |  | United Arab Emirates | Yes |  |  |
| 59 | Abdalhamid, B. [57] | 2018 | Case report |  | Saudi Arabia | Yes |  |  |
| 60 | Schwartz, I. S. [58] | 2017 | Case report |  | Canada | Yes |  |  |
| 61 | Mohsin, J. [59] | 2017 | Case report | 2016, 2017 | Oman | Yes |  |  |
| 62 | Choi, H. I. [60] | 2017 | Case report | 2013 | Korea | Yes |  |  |
| 63 | Biswal, M. [61] | 2017 | Case report | 2017 | India | Yes |  |  |
| 64 | Kim, T. H. [62] | 2016 | Case report | 2010-2013 | Korea | Yes |  |  |
| 65 | Kumar, D. [63] | 2015 | Case report |  | India | Yes |  |  |
| 66 | Emara, M. [64] | 2015 | Case report | 2014 | Kuwait | Yes |  |  |
| 67 | Satoh, K. [65] | 2009 | Case report |  | Japan | Yes |  |  |

Note: ID 40-67 are studies with sample size smaller than 5.

**References:**

1. Bajpai V, Govindaswamy A, Sagar S, Kumar S, Garg P, Xess I et al. Multidrug-Resistant Candida auris Fungemia in Critical Care Units: Experience from a Tertiary Care Hospital in India. Microb Drug Resist. 2019. doi:10.1089/mdr.2019.0021.

2. van Schalkwyk E, Mpembe RS, Thomas J, Shuping L, Ismail H, Lowman W et al. Epidemiologic Shift in Candidemia Driven by Candida auris, South Africa, 2016-2017(1). Emerging infectious diseases. 2019;25(9):1698-707. doi:10.3201/eid2509.190040.

3. Chibabhai V, Fadana V, Bosman N, Nana T. Comparative sensitivity of 1,3 beta-D-glucan for common causes of candidaemia in South Africa. Mycoses. 2019. doi:10.1111/myc.12982.

4. Sana F, Hussain W, Zaman G, LuqmanSatti, UmerKhurshid, Khadim MT. Candida auris associated outbreak report from Pakistan: a success story of infection control in ICU of a tertiary care hospital. The Journal of hospital infection. 2019. doi:10.1016/j.jhin.2019.06.011.

5. Iguchi S, Itakura Y, Yoshida A, Kamada K, Mizushima R, Arai Y et al. Candida auris: A pathogen difficult to identify, treat, and eradicate and its characteristics in Japanese strains. J Infect Chemother. 2019. doi:10.1016/j.jiac.2019.05.034.

6. Hamprecht A, Barber AE, Mellinghoff SC, Thelen P, Walther G, Yu Y et al. Candida auris in Germany and Previous Exposure to Foreign Healthcare. Emerging infectious diseases. 2019;25(9). doi:10.3201/eid2509.190262.

7. Ceballos-Garzon A, Cortes G, Morio F, Zamora-Cruz EL, Linares MY, Ariza BE et al. Comparison between MALDI-TOF MS and MicroScan in the identification of emerging and multidrug resistant yeasts in a fourth-level hospital in Bogota, Colombia. BMC Microbiol. 2019;19(1):106. doi:10.1186/s12866-019-1482-y.

8. Adam RD, Revathi G, Okinda N, Fontaine M, Shah J, Kagotho E et al. Analysis of Candida auris fungemia at a single facility in Kenya. International journal of infectious diseases : IJID : official publication of the International Society for Infectious Diseases. 2019. doi:10.1016/j.ijid.2019.06.001.

9. Sayeed MA, Farooqi J, Jabeen K, Awan S, Mahmood SF. Clinical spectrum and factors impacting outcome of Candida auris: a single center study from Pakistan. BMC Infect Dis. 2019;19(1):384. doi:10.1186/s12879-019-3999-y.

10. Ruiz-Gaitan A, Martinez H, Moret AM, Calabuig E, Tasias M, Alastruey-Izquierdo A et al. Detection and treatment of Candida auris in an outbreak situation: risk factors for developing colonization and candidemia by this new species in critically ill patients. Expert review of anti-infective therapy. 2019;17(4):295-305. doi:10.1080/14787210.2019.1592675.

11. Park JY, Bradley N, Brooks S, Burney S, Wassner C. Management of Patients with Candida auris Fungemia at Community Hospital, Brooklyn, New York, USA, 2016-2018(1). Emerging infectious diseases. 2019;25(3):601-2. doi:10.3201/eid2503.180927.

12. Kwon YJ, Shin JH, Byun SA, Choi MJ, Won EJ, Lee D et al. Candida auris Clinical Isolates from South Korea: Identification, Antifungal Susceptibility, and Genotyping. Journal of clinical microbiology. 2019;57(4). doi:10.1128/jcm.01624-18.

13. Jung J, Kim MJ, Kim JY, Lee JY, Kwak SH, Hong MJ et al. Candida auris colonization or infection of the ear: A single-center study in South Korea from 2016 to 2018. Medical mycology. 2019. doi:10.1093/mmy/myz020.

14. Escandon P, Chow NA, Caceres DH, Gade L, Berkow EL, Armstrong P et al. Molecular Epidemiology of Candida auris in Colombia Reveals a Highly Related, Countrywide Colonization With Regional Patterns in Amphotericin B Resistance. Clinical infectious diseases : an official publication of the Infectious Diseases Society of America. 2019;68(1):15-21. doi:10.1093/cid/ciy411.

15. Barantsevich NE, Orlova OE, Shlyakhto EV, Johnson EM, Woodford N, Lass-Floerl C et al. Emergence of Candida auris in Russia. The Journal of hospital infection. 2019. doi:10.1016/j.jhin.2019.02.021.

16. Tian S, Rong C, Nian H, Li F, Chu Y, Cheng S et al. First cases and risk factors of super yeast Candida auris infection or colonization from Shenyang, China. Emerging microbes & infections. 2018;7(1):128. doi:10.1038/s41426-018-0131-0.

17. Ruiz-Gaitan A, Moret AM, Tasias-Pitarch M, Aleixandre-Lopez AI, Martinez-Morel H, Calabuig E et al. An outbreak due to Candida auris with prolonged colonisation and candidaemia in a tertiary care European hospital. Mycoses. 2018;61(7):498-505. doi:10.1111/myc.12781.

18. Kohlenberg A, Struelens MJ, Monnet DL, Plachouras D. Candida auris: epidemiological situation, laboratory capacity and preparedness in European Union and European Economic Area countries, 2013 to 2017. Euro surveillance : bulletin Europeen sur les maladies transmissibles = European communicable disease bulletin. 2018;23(13). doi:10.2807/1560-7917.es.2018.23.13.18-00136.

19. Khan Z, Ahmad S, Al-Sweih N, Joseph L, Alfouzan W, Asadzadeh M. Increasing prevalence, molecular characterization and antifungal drug susceptibility of serial Candida auris isolates in Kuwait. PloS one. 2018;13(4):e0195743. doi:10.1371/journal.pone.0195743.

20. Khan Z, Ahmad S, Benwan K, Purohit P, Al-Obaid I, Bafna R et al. Invasive Candida auris infections in Kuwait hospitals: epidemiology, antifungal treatment and outcome. Infection. 2018;46(5):641-50. doi:10.1007/s15010-018-1164-y.

21. Govender NP, Magobo RE, Mpembe R, Mhlanga M, Matlapeng P, Corcoran C et al. Candida auris in South Africa, 2012-2016. Emerging infectious diseases. 2018;24(11):2036-40. doi:10.3201/eid2411.180368.

22. Eyre DW, Sheppard AE, Madder H, Moir I, Moroney R, Quan TP et al. A Candida auris Outbreak and Its Control in an Intensive Care Setting. The New England journal of medicine. 2018;379(14):1322-31. doi:10.1056/NEJMoa1714373.

23. Escandon P, Caceres DH, Espinosa-Bode A, Rivera S, Armstrong P, Vallabhaneni S et al. Notes from the Field: Surveillance for Candida auris - Colombia, September 2016-May 2017. MMWR Morbidity and mortality weekly report. 2018;67(15):459-60. doi:10.15585/mmwr.mm6715a6.

24. Chowdhary A, Prakash A, Sharma C, Kordalewska M, Kumar A, Sarma S et al. A multicentre study of antifungal susceptibility patterns among 350 Candida auris isolates (2009-17) in India: role of the ERG11 and FKS1 genes in azole and echinocandin resistance. The Journal of antimicrobial chemotherapy. 2018;73(4):891-9. doi:10.1093/jac/dkx480.

25. Chow NA, Gade L, Tsay SV, Forsberg K, Greenko JA, Southwick KL et al. Multiple introductions and subsequent transmission of multidrug-resistant Candida auris in the USA: a molecular epidemiological survey. The Lancet Infectious diseases. 2018;18(12):1377-84. doi:10.1016/s1473-3099(18)30597-8.

26. Arauz AB, Caceres DH, Santiago E, Armstrong P, Arosemena S, Ramos C et al. Isolation of Candida auris from 9 patients in Central America: Importance of accurate diagnosis and susceptibility testing. Mycoses. 2018;61(1):44-7. doi:10.1111/myc.12709.

27. Adams E, Quinn M, Tsay S, Poirot E, Chaturvedi S, Southwick K et al. Candida auris in Healthcare Facilities, New York, USA, 2013-2017. Emerging infectious diseases. 2018;24(10):1816-24. doi:10.3201/eid2410.180649.

28. Ben-Ami R, Berman J, Novikov A, Bash E, Shachor-Meyouhas Y, Zakin S et al. Multidrug-Resistant Candida haemulonii and C. auris, Tel Aviv, Israel. Emerging infectious diseases. 2017;23(1). doi:10.3201/eid2302.161486.

29. Rudramurthy SM, Chakrabarti A, Paul RA, Sood P, Kaur H, Capoor MR et al. Candida auris candidaemia in Indian ICUs: analysis of risk factors. The Journal of antimicrobial chemotherapy. 2017;72(6):1794-801. doi:10.1093/jac/dkx034.

30. Morales-Lopez SE, Parra-Giraldo CM, Ceballos-Garzon A, Martinez HP, Rodriguez GJ, Alvarez-Moreno CA et al. Invasive Infections with Multidrug-Resistant Yeast Candida auris, Colombia. Emerging infectious diseases. 2017;23(1):162-4. doi:10.3201/eid2301.161497.

31. Al-Siyabi T, Al Busaidi I, Balkhair A, Al-Muharrmi Z, Al-Salti M, Al'Adawi B. First report of Candida auris in Oman: Clinical and microbiological description of five candidemia cases. The Journal of infection. 2017;75(4):373-6. doi:10.1016/j.jinf.2017.05.016.

32. Schelenz S, Hagen F, Rhodes JL, Abdolrasouli A, Chowdhary A, Hall A et al. First hospital outbreak of the globally emerging Candida auris in a European hospital. Antimicrobial resistance and infection control. 2016;5:35. doi:10.1186/s13756-016-0132-5.

33. Prakash A, Sharma C, Singh A, Kumar Singh P, Kumar A, Hagen F et al. Evidence of genotypic diversity among Candida auris isolates by multilocus sequence typing, matrix-assisted laser desorption ionization time-of-flight mass spectrometry and amplified fragment length polymorphism. Clinical microbiology and infection : the official publication of the European Society of Clinical Microbiology and Infectious Diseases. 2016;22(3):277.e1-9. doi:10.1016/j.cmi.2015.10.022.

34. Calvo B, Melo AS, Perozo-Mena A, Hernandez M, Francisco EC, Hagen F et al. First report of Candida auris in America: Clinical and microbiological aspects of 18 episodes of candidemia. The Journal of infection. 2016;73(4):369-74. doi:10.1016/j.jinf.2016.07.008.

35. Chowdhary A, Anil Kumar V, Sharma C, Prakash A, Agarwal K, Babu R et al. Multidrug-resistant endemic clonal strain of Candida auris in India. European journal of clinical microbiology & infectious diseases : official publication of the European Society of Clinical Microbiology. 2014;33(6):919-26. doi:10.1007/s10096-013-2027-1.

36. Sarma S, Kumar N, Sharma S, Govil D, Ali T, Mehta Y et al. Candidemia caused by amphotericin B and fluconazole resistant Candida auris. Indian journal of medical microbiology. 2013;31(1):90-1. doi:10.4103/0255-0857.108746.

37. Chowdhary A, Sharma C, Duggal S, Agarwal K, Prakash A, Singh PK et al. New clonal strain of Candida auris, Delhi, India. Emerging infectious diseases. 2013;19(10):1670-3. doi:10.3201/eid1910.130393.

38. Ding CH, Situ SF, Steven A, Razak MFA. The Pitfall of Utilizing a Commercial Biochemical Yeast Identification Kit to Detect Candida auris. Ann Clin Lab Sci. 2019;49(4):546-9.

39. Crea F, Codda G, Orsi A, Battaglini A, Giacobbe DR, Delfino E et al. Isolation of Candida auris from invasive and non-invasive samples of a patient suffering from vascular disease, Italy, July 2019. Euro surveillance : bulletin Europeen sur les maladies transmissibles = European communicable disease bulletin. 2019;24(37). doi:10.2807/1560-7917.ES.2019.24.37.1900549.

40. Vogelzang EH, Weersink AJL, van Mansfeld R, Chow NA, Meis JF, van Dijk K. The First Two Cases of Candida auris in The Netherlands. Journal of fungi (Basel, Switzerland). 2019;5(4). doi:10.3390/jof5040091.

41. Supreeth S, Al Ghafri KA, Kumar JR, Al Balushi ZY. First report of Candia auris spondylodiscitis in Oman - A rare presentation. World Neurosurg. 2019. doi:10.1016/j.wneu.2019.09.021.

42. Stathi A, Loukou I, Kirikou H, Petrocheilou A, Moustaki M, Velegraki A et al. Isolation of Candida auris from cystic fibrosis patient, Greece, April 2019. Euro surveillance : bulletin Europeen sur les maladies transmissibles = European communicable disease bulletin. 2019;24(29). doi:10.2807/1560-7917.ES.2019.24.29.1900400.

43. Elsawy A, Alquthami K, Alkhutani N, Marwan D, Abbas A. The second confirmed case of Candida auris from Saudi Arabia. Journal of infection and public health. 2019. doi:10.1016/j.jiph.2019.07.011.

44. Tang HJ, Lai CC, Lai FJ, Li SY, Liang HY, Hsueh PR. Emergence of multidrug-resistant Candida auris in Taiwan. International journal of antimicrobial agents. 2019. doi:10.1016/j.ijantimicag.2019.02.011.

45. O'Connor C, Bicanic T, Dave J, Evans TJ, Moxey P, Adamu U et al. Candida auris outbreak on a vascular ward - the unexpected arrival of an anticipated pathogen. The Journal of hospital infection. 2019. doi:10.1016/j.jhin.2019.06.002.

46. Abastabar M, Haghani I, Ahangarkani F, Rezai MS, Taghizadeh Armaki M, Roodgari S et al. Candida auris otomycosis in Iran and review of recent literature. Mycoses. 2019;62(2):101-5. doi:10.1111/myc.12886.

47. Heath CH, Dyer JR, Pang S, Coombs GW, Gardam DJ. Candida auris Sternal Osteomyelitis in a Man from Kenya Visiting Australia, 2015. Emerging infectious diseases. 2019;25(1):192-4. doi:10.3201/eid2501.181321.

48. Wang X, Bing J, Zheng Q, Zhang F, Liu J, Yue H et al. The first isolate of Candida auris in China: clinical and biological aspects. Emerging microbes & infections. 2018;7(1):93. doi:10.1038/s41426-018-0095-0.

49. Tan YE, Tan AL. Arrival of Candida auris Fungus in Singapore: Report of the First 3 Cases. Annals of the Academy of Medicine, Singapore. 2018;47(7):260-2.

50. Riat A, Neofytos D, Coste A, Harbarth S, Bizzini A, Grandbastien B et al. First case of Candida auris in Switzerland: discussion about preventive strategies. Swiss medical weekly. 2018;148:w14622. doi:10.4414/smw.2018.14622.

51. Pekard-Amenitsch S, Schriebl A, Posawetz W, Willinger B, Kolli B, Buzina W. Isolation of Candida auris from Ear of Otherwise Healthy Patient, Austria, 2018. Emerging infectious diseases. 2018;24(8):1596-7. doi:10.3201/eid2408.180495.

52. Parra-Giraldo CM, Valderrama SL, Cortes-Fraile G, Garzon JR, Ariza BE, Morio F et al. First report of sporadic cases of Candida auris in Colombia. International journal of infectious diseases : IJID : official publication of the International Society for Infectious Diseases. 2018;69:63-7. doi:10.1016/j.ijid.2018.01.034.

53. Mohd Tap R, Lim TC, Kamarudin NA, Ginsapu SJ, Abd Razak MF, Ahmad N et al. A Fatal Case of Candida auris and Candida tropicalis Candidemia in Neutropenic Patient. Mycopathologia. 2018;183(3):559-64. doi:10.1007/s11046-018-0244-y.

54. Chen Y, Zhao J, Han L, Qi L, Fan W, Liu J et al. Emergency of fungemia cases caused by fluconazole-resistant Candida auris in Beijing, China. The Journal of infection. 2018;77(6):561-71. doi:10.1016/j.jinf.2018.09.002.

55. Belkin A, Gazit Z, Keller N, Ben-Ami R, Wieder-Finesod A, Novikov A et al. Candida auris Infection Leading to Nosocomial Transmission, Israel, 2017. Emerging infectious diseases. 2018;24(4):801-4. doi:10.3201/eid2404.171715.

56. Alatoom A, Sartawi M, Lawlor K, AbdelWareth L, Thomsen J, Nusair A et al. Persistent candidemia despite appropriate fungal therapy: First case of Candida auris from the United Arab Emirates. International journal of infectious diseases : IJID : official publication of the International Society for Infectious Diseases. 2018;70:36-7. doi:10.1016/j.ijid.2018.02.005.

57. Abdalhamid B, Almaghrabi R, Althawadi S, Omrani A. First report of Candida auris infections from Saudi Arabia. Journal of infection and public health. 2018;11(4):598-9. doi:10.1016/j.jiph.2018.05.010.

58. Schwartz IS, Hammond GW. First reported case of multidrug-resistant Candida auris in Canada. Canada communicable disease report = Releve des maladies transmissibles au Canada. 2017;43(7-8):150-3.

59. Mohsin J, Hagen F, Al-Balushi ZAM, de Hoog GS, Chowdhary A, Meis JF et al. The first cases of Candida auris candidaemia in Oman. Mycoses. 2017;60(9):569-75. doi:10.1111/myc.12647.

60. Choi HI, An J, Hwang JJ, Moon SY, Son JS. Otomastoiditis caused by Candida auris: Case report and literature review. Mycoses. 2017;60(8):488-92. doi:10.1111/myc.12617.

61. Biswal M, Rudramurthy SM, Jain N, Shamanth AS, Sharma D, Jain K et al. Controlling a possible outbreak of Candida auris infection: lessons learnt from multiple interventions. The Journal of hospital infection. 2017;97(4):363-70. doi:10.1016/j.jhin.2017.09.009.

62. Kim TH, Kweon OJ, Kim HR, Lee MK. Identification of Uncommon Candida Species Using Commercial Identification Systems. J Microbiol Biotechnol. 2016;26(12):2206-13. doi:10.4014/jmb.1609.09012.

63. Kumar D, Banerjee T, Pratap CB, Tilak R. Itraconazole-resistant Candida auris with phospholipase, proteinase and hemolysin activity from a case of vulvovaginitis. Journal of infection in developing countries. 2015;9(4):435-7. doi:10.3855/jidc.4582.

64. Emara M, Ahmad S, Khan Z, Joseph L, Al-Obaid I, Purohit P et al. Candida auris candidemia in Kuwait, 2014. Emerging infectious diseases. 2015;21(6):1091-2. doi:10.3201/eid2106.150270.

65. Satoh K, Makimura K, Hasumi Y, Nishiyama Y, Uchida K, Yamaguchi H. Candida auris sp. nov., a novel ascomycetous yeast isolated from the external ear canal of an inpatient in a Japanese hospital. Microbiology and immunology. 2009;53(1):41-4. doi:10.1111/j.1348-0421.2008.00083.x.
