# Supplementary material for: Is the superbug fungus really so scary? A systematic review and meta-analysis of global epidemiology and mortality of Candida auris
Source: BMC Infect Dis. 2020 Nov 11;20:827. doi: 10.1186/s12879-020-05543-0 (PMC7656719; doi:10.1186/s12879-020-05543-0)
Supplement: Supplementary file 2 — Additional file 2: Table S2 Quality assessment of the studies included in the meta-analysis for mortality and drug resistance patterns. [file 12879_2020_5543_MOESM2_ESM.docx]

Table S2 Quality assessment of the studies included in the meta-analysis for mortality and drug resistance patterns

| ID | Author | Publication Year | Analysis for drug resistance | Analysis for mortality | Quality score | Article quality |
| --- | --- | --- | --- | --- | --- | --- |
| 1 | van Schalkwyk, E. | 2019 |  | Yes | 7 | Moderate |
| 2 | Sana, F. | 2019 |  | Yes | 7 | Moderate |
| 3 | Iguchi, S. | 2019 | Yes |  | 6 | Moderate |
| 4 | Hamprecht, A. | 2019 | Yes | Yes | 5 | Moderate |
| 5 | Ceballos-Garzon, A. | 2019 | Yes |  | 6 | Moderate |
| 6 | Adam, R. D. | 2019 | Yes | Yes | 7 | Moderate |
| 7 | Sayeed, M. A. | 2019 | Yes | Yes | 6 | Moderate |
| 8 | Ruiz-Gaitan, A. | 2019 |  | Yes | 7 | Moderate |
| 9 | Kwon, Y. J. | 2019 | Yes |  | 6 | Moderate |
| 10 | Barantsevich, N. E. | 2019 | Yes | Yes | 6 | Moderate |
| 11 | Tian, S. | 2018 | Yes |  | 6 | Moderate |
| 12 | Ruiz-Gaitan, A. | 2018 | Yes |  | 7 | Moderate |
| 13 | Khan, Z. | 2018 |  | Yes | 6 | Moderate |
| 14 | Khan, Z. | 2018 | Yes |  | 6 | Moderate |
| 15 | Eyre, D. W. | 2018 | Yes | Yes | 9 | High |
| 16 | Chowdhary, A. | 2018 | Yes |  | 6 | Moderate |
| 17 | Chow, N. A. | 2018 | Yes |  | 6 | Moderate |
| 18 | Arauz, A. B. | 2018 | Yes | Yes | 6 | Moderate |
| 19 | Adams, E. | 2018 | Yes | Yes | 6 | Moderate |
| 20 | Ben-Ami, R. | 2017 | Yes | Yes | 5 | Moderate |
| 21 | Rudramurthy, S. M. | 2017 | Yes | Yes | 7 | Moderate |
| 22 | Morales-Lopez, S. E. | 2017 | Yes | Yes | 6 | Moderate |
| 23 | Al-Siyabi, T. | 2017 | Yes | Yes | 6 | Moderate |
| 24 | Schelenz, S. | 2016 | Yes |  | 7 | Moderate |
| 25 | Prakash, A. | 2016 |  |  | 6 | Moderate |
| 26 | Calvo, B. | 2016 | Yes | Yes | 5 | Moderate |
| 27 | Chowdhary, A. | 2014 |  | Yes | 6 | Moderate |
| 28 | Sarma, S. | 2013 |  | Yes | 5 | Moderate |
| 29 | Chowdhary, A. | 2013 |  | Yes | 6 | Moderate |
