# Supplementary figures and images for: Is the superbug fungus really so scary? A systematic review and meta-analysis of global epidemiology and mortality of Candida auris
Source: BMC Infect Dis. 2020 Nov 11;20:827. doi: 10.1186/s12879-020-05543-0 (PMC7656719; doi:10.1186/s12879-020-05543-0)

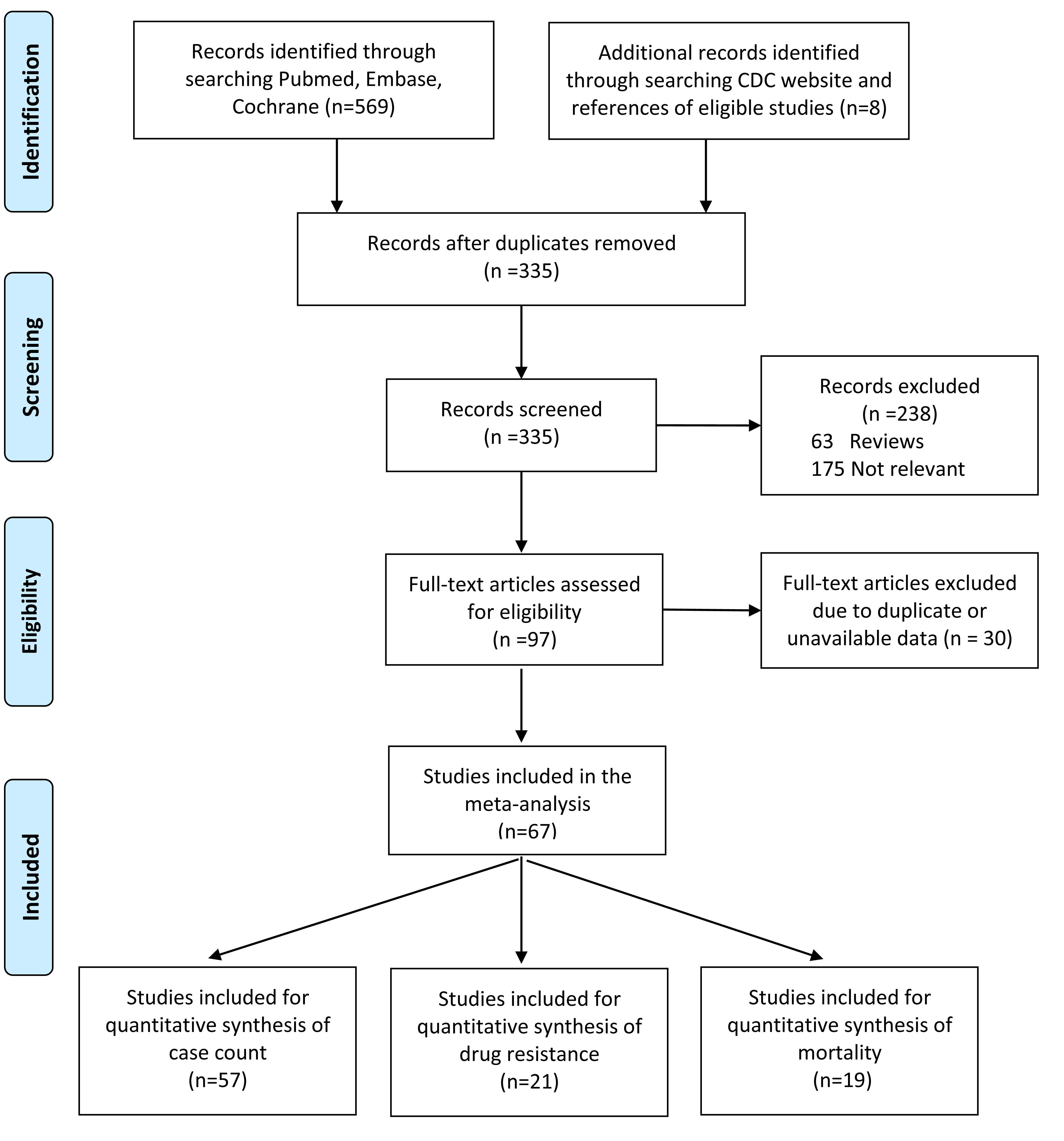

Supplement: Supplementary file 3 — Additional file 3: Figure S1 Flowchart showing study search and selection. [file 12879_2020_5543_MOESM3_ESM.tif]

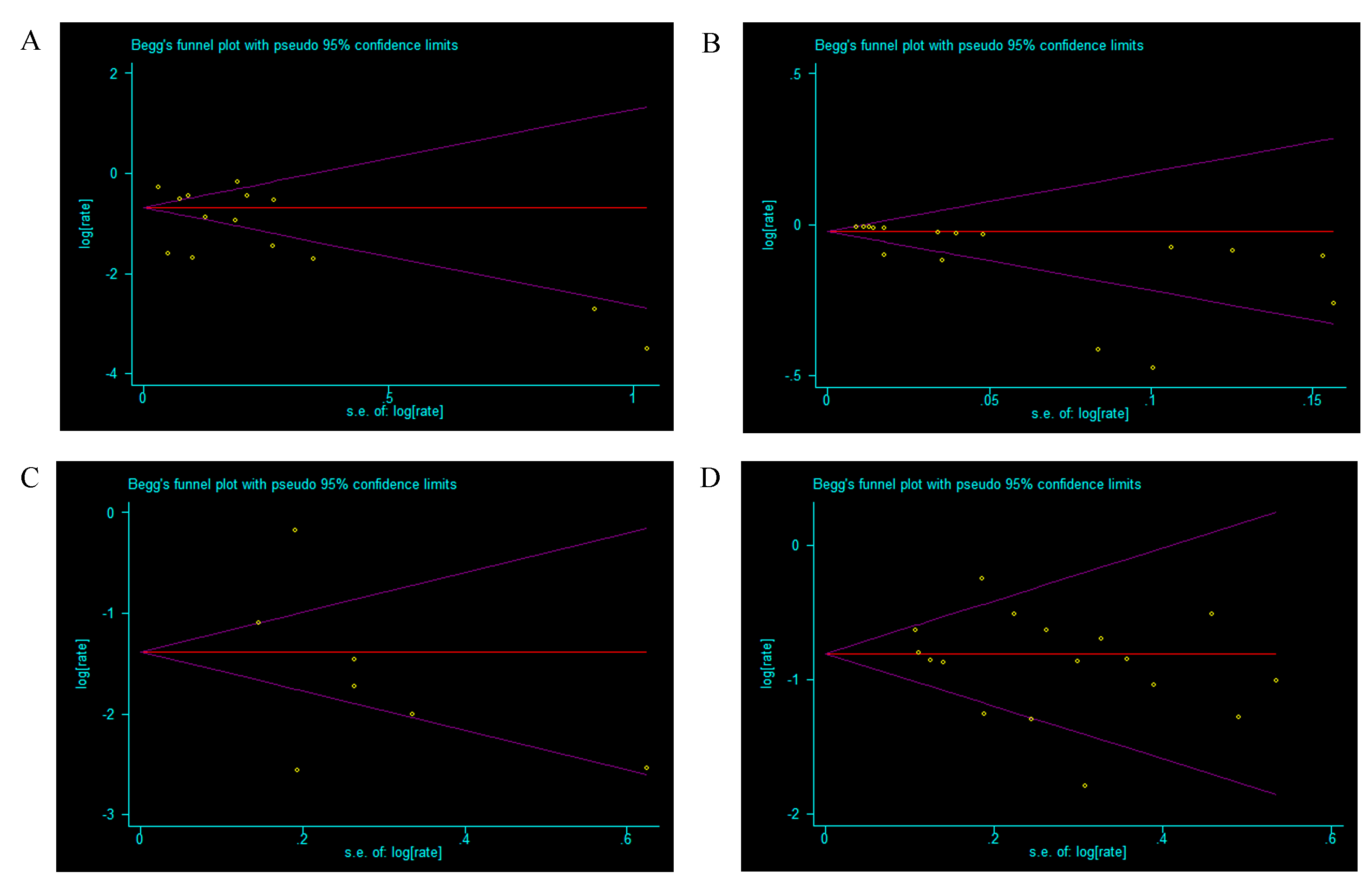

Supplement: Supplementary file 4 — Additional file 4: Figure S2 Funnel plot for BSI rate of C. auris (A), drug resistance of C. auris to fluconazole (B) and amphotericin B (C), crude mortality (D). [file 12879_2020_5543_MOESM4_ESM.tif]

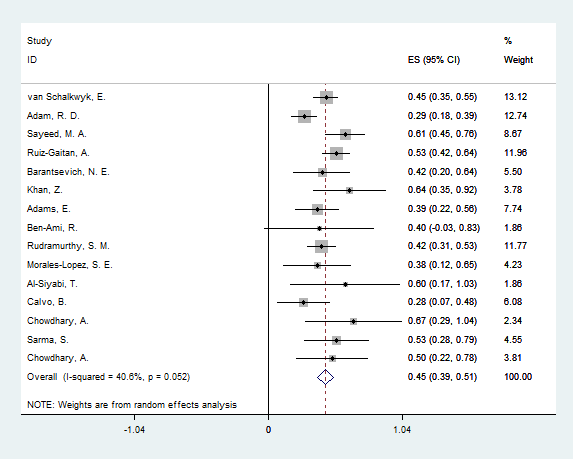

Supplement: Supplementary file 5 — Additional file 5: Figure S3 Forest plot on the crude mortality for BSI of C. auris. [file 12879_2020_5543_MOESM5_ESM.tif]
